# Use of Thyroid Hormones in Hypothyroid and Euthyroid Patients: A 2020 THESIS Questionnaire Survey of Members of the Swedish Endocrine Society

**DOI:** 10.3389/fendo.2021.795111

**Published:** 2021-12-06

**Authors:** Tereza Planck, Mikael Lantz, Petros Perros, Enrico Papini, Roberto Attanasio, Endre V. Nagy, Laszlo Hegedüs

**Affiliations:** ^1^ Department of Endocrinology, Skåne University Hospital, Malmö, Sweden; ^2^ Department of Clinical Sciences Malmö, Lund University, Lund, Sweden; ^3^ Department of Endocrinology, Royal Victoria Infirmary, Newcastle upon Tyne, United Kingdom; ^4^ Department of Endocrinology and Metabolism, Regina Apostolorum Hospital, Rome, Italy; ^5^ Endocrine Unit, Institutes for Care and Scientific Research (IRCCS) Orthopedic Institute Galeazzi, Milan, Italy; ^6^ Division of Endocrinology, Department of Medicine, Faculty of Medicine, University of Debrecen, Debrecen, Hungary; ^7^ Department of Endocrinology and Metabolism, Odense University Hospital, Odense, Denmark

**Keywords:** levothyroxine, liothyronine, desiccated thyroid extract (DTE), hypothyroidism, euthyroidism, survey, Swedish Endocrine Society

## Abstract

**Background:**

The standard treatment of hypothyroidism is levothyroxine (LT-4). However, there are several controversies regarding treatment of hypothyroid patients.

**Aim:**

To investigate the Swedish endocrinologists’ use of thyroid hormones in hypothyroid and euthyroid individuals.

**Methods:**

Physician members of the Swedish Endocrine Society (SEF) were invited by e-mail to participate in an online survey investigating this topic.

**Results:**

Out of the eligible 411 members, 116 (28.2%) responded. The majority (98.9%) stated that L-T4 is the treatment of choice. However, around 50% also prescribed liothyronine (L-T3) or a combination of L-T4+L-T3 in their practice. Combination therapy was mostly (78.5%) used in patients with persistent hypothyroid symptoms despite biochemical euthyroidism on L-T4 treatment. Most respondents prescribed L-T4 tablets and did not expect any major changes with alternative formulations such as soft-gel capsules or liquid formulations in situations influencing the bioavailability of L-T4. In euthyroid patients, 49.5% replied that treatment with thyroid hormones was never indicated, while 47.3% would consider L-T4 for euthyroid infertile women with high thyroid peroxidase (TPO) antibody levels.

**Conclusion:**

The treatment of choice for hypothyroidism in Sweden is L-T4 tablets. Combination therapy with L-T4+L-T3 tablets was considered for patients with persistent symptoms despite biochemical euthyroidism. Soft-gel capsules and liquid solutions of L-T4 were infrequently prescribed. Swedish endocrinologists’ deviation from endocrine society guidelines merits further study.

## Introduction

Hypothyroidism, overt or subclinical, affects approximately 5% of the adult population (1). The standard treatment for hypothyroidism is L-thyroxine (L-T4) and treatment is monitored by measuring the serum levels of thyroid-stimulating hormone (TSH) (2). Different L-T4 formulations are commercially available, including tablets, soft-gel capsules, and liquid solutions. Soft-gel capsules and liquid solutions were introduced to overcome potential problems due to intolerance and bioavailability, as the latter may be influenced by L-T4 administration together with foods and beverages or certain medications, and by the presence of concurrent gastrointestinal diseases (3, 4).

In Sweden, only L-T4 tablets were widely available at the time of the survey whereas other formulations could only be obtained with an individual application by the physician and approval by the Swedish Medical Products Agency (*Läkemedelsverket*). As in other countries (5, 6), the number of L-T4 prescriptions has recently increased in Sweden (7). The reason for this seems to be a decreasing TSH threshold for initiating of treatment for hypothyroidism (8–10) and an increase in disease awareness by both physicians and patients.

Approximately 10% of patients treated with L-T4 for hypothyroidism report persisting hypothyroid symptoms despite biochemical euthyroidism (11). Several hypotheses have been proposed to explain this phenomenon; they include individual variation in the ability to transport L-T4 and synthesize triiodothyronine from L-T4 in peripheral tissues of hypothyroid patients treated with L-T4, possibly leading to intracellular hypothyroidism in one or several tissues and incomplete resolution of symptoms [reviewed in (12)]. According to this hypothesis, persistent symptoms could be relieved by adding synthetic triiodothyronine, liothyronine (L-T3). Many unsubstantiated claims of the positive effects of L-T3 treatment are aired online and *via* patient organizations, resulting in an increasing number of patients requesting treatment with L-T3 (13, 14). As a consequence the L-T3 prescriptions have increased dramatically worldwide, including in Sweden (7). To date, a number of randomized controlled trials have studied the effect of combined L-T4+L-T3 and five systemic reviews/meta-analyses have concluded that the combined treatment is not superior to standard L-T4 treatment with respect to hypothyroid symptoms and quality of life (15–18) or patient preference (19). Data on long-term safety are limited (20, 21). Therefore, European guidelines restrict the recommendation of L-T3 to that of experimental treatment (2). Desiccated thyroid extract (DTE), based on porcine thyroid and containing both L-T4 and L-T3, among other substances, is, in Sweden, only available with an individual license approved by the Swedish Medical Products Agency due to limited scientific evidence for its benefit (22, 23).

Sweden does not have national guidelines for treatment of hypothyroidism but local guidelines generally follow international recommendations and are a result of a collaboration between endocrinologists and general practitioners. Most Swedish patients with hypothyroidism are treated by general practitioners. Patients referred to endocrinologists include those with concomitant other thyroid or endocrine disorders treated by endocrinologists, some pregnant patients or patients preparing for or undergoing treatment for infertility, and complicated cases of hypothyroidism.

This survey is part of an ongoing international initiative referred to as THESIS (Treatment of Hypothyroidism in Europe by Specialists: An International Survey) investigating current attitudes of European endocrinologists towards the treatment of hypothyroidism in 28 countries. To date, the Italian (24), Bulgarian (25), Romanian (26), Polish (27), Spanish (28), and Danish (29) national surveys have been published. The aim was to examine the opinions of members of the Swedish Endocrine Society (Svenska Endokrinologföreningen, SEF) on the treatment of hypothyroid and euthyroid patients with thyroid hormones.

## Material and Methods

### Survey

We used a web-based survey constructed with Survey Monkey, an open-access platform that provides various questionnaire templates. The survey was developed in English to ensure comparability between respondents from different European countries and consisted of 23 questions, which could be completed in 10-15 minutes. Eight questions on demographic data (A1-A8) were followed by 23 questions on the practice of treating hypothyroid and euthyroid patients (B1-B23). Space for free text was available at the end of the questionnaire (B24). The survey questionnaire is presented in **Supplement 1**.

An invitation e-mail containing the description of the study, including an electronic link leading to the questionnaire, was sent to the 411 members of the SEF registered as physicians (nurses, medical students, and other professional categories were excluded) on 19 November 2020, followed by two reminders on 4 December 2020 and 14 January 2021, respectively. The survey closed on 31 January 2021. Anonymized survey responses were collected and electronically stored by the Survey Monkey service. Repeat submissions from the same IP-address were automatically blocked.

### Statistical Analysis

Only data from respondents who had completed all questions about demographic data were considered valid for statistical analysis. In all analyses, respondents who did not know the answer to a question were pooled with respondents who did not provide an answer to that question. The Pearson goodness of fit chi^2^-test was used to compare frequencies between the categorical variables. Pearson chi^2^-test was used to test if variables in the demographic data (section A) were independent of the outcome in questions in section B. If any variable was not independent of the outcome in any question in section B, a logistic regression analysis was done. A two-sided p-value of <0.05 was considered statistically significant. All analyses were performed using IBM SPSS statistics software version 26 (SPSS Inc., Chicago, IL, USA).

## Results

### Sample Characteristics

One hundred sixteen (28.2%) of the eligible members of SEF responded and 93 (22.6%) completed all the questions of the survey. The demographic data of the respondents are summarized in **Table 1**. All respondents had at least one medical specialty; 106 (91.4%) were endocrinologists.

**Table 1:** Characteristics of the 116 respondents.

	N (%)
**Gender**	
Male	61 (52.6)
Female	55 (47.4)
**Age**	
20-30	2 (1.7)
31-40	24 (20.7)
41-50	35 (30.2)
51-60	26 (22.4)
61-70	21 (18.1)
70+	8 (6.9)
**Years in medical practice**	
0-10	10 (8.6)
11-20	49 (42.2)
21-30	29 (25.0)
31-40	15 (12.9)
40+	13 (11.2)
Specialization*	
Endocrinology	106 (91.4)
Internal medicine	86 (74.1)
Other	4 (3.4)
Society mebership*	
SEF	116 (100)
ETA	10 (8.6)
ATA	1 (0.9)
Additional national scientific societies	80 (69.0)
Place of practice*	
University centre	55 (47.4)
Regional hospital	58 (50.0)
Private clinic	4 (3.4)
General practice	1 (0.9)
Specialist practice	9 (7.8)
**Clinically active**	
Yes	109 (94.0)
No	7 (6.0)
**Management of patients with thyroid disease**	
Daily	57 (49.1)
Weekly	49 (42.2)
Rarely	10 (8.6)
**Management of patients with hypothyroidism**	
>100 patients/year	18 (15.5)
51-100 patients/year	34 (29.3)
10-50 patients/year	57 (49.1)
Rarely	7 (6.0)

SEF, Swedish Endocrine Society; ETA, European Thyroid Association; ATA, American Thyroid Association.

*Total is >100% as responders were allowed to tick more than one options in the questionnaire.

### Treatment of Choice for Hypothyroidism

Concerning the first choice for treatment of hypothyroidism, the vast majority (92; 79.3% corresponding to 98.9% of those who responded to the question) stated that their preference was L-T4. One (0.9%) suggested L-T4 and L-T3 combination therapy, while no responder chose L-T3 monotherapy or DTE. Although combination therapy was not the initial treatment of choice for nearly all respondents, a significant proportion of responders who answered this question used L-T3-containing treatments in their clinical practice for specific indications [48 (51.6%) L-T3, 46 (49.5%) L-T4 + L-T3 combination, 16 (17.2%) DTE].

### Use of Different L-T4 Formulations

The majority of those who answered this question indicated that the formulation of L-T4 they recommended was the one dispensed (83, 89.2%), 2 (2.2%) that they had control over the type of L-T4 dispensed but had to justify it to the regulatory authorities. Finally, 5 (5.4%) had no control, and 3 (3.2%) stated that general practitioners mostly chose the dispensed type of L-T4 (L-T4 dispensed as recommended + need to justify to authorities vs. no control + chosen by general practitioners; p<0.001). **Table 2** shows the respondents’ preferences for different L-T4 formulations in several clinical scenarios. The majority preferred tablets and did not expect any major effect of switching to a different formulation such as liquid L-T4 or soft-gel capsules, or tablets from a different manufacturer in these situations (p<0.001).

**Table 2:** Use of different L-T4 formulations in various clinical situations.

Question	I expect no major changes with the different formulations, n (%)	Tablets/tablets from another manufacturer, n (%)	Soft-gel capsules, n (%)	Liquid solutions, n (%)
B5- Interfering drugs may influence the stability of therapy. Which L-T4 preparation is in your experience least likely to be subject to variable absorption?*	53 (57.0)	30 (32.3)	8 (8.6)	2 (2.2)
B6- Which of the following preparations of L-T4 would you prescribe in case of first diagnosis of hypothyroidism when the patient self-reports intolerance to various foods raising the possibility of celiac disease, malabsorption, lactose intolerance, or intolerance to common excipients?*	24 (25.5)	61 (65.6)	7 (7.5)	1 (1.1)
B7- Which of the following preparations of L-T4 would you prescribe for a patient established on L-T4 who has unexplained poor biochemical control of hypothyroidism?*	30 (32.3)	49 (52.7)	12 (12.9)	2 (2.2)
B8- Which of the following preparations of L-T4 would you prescribe for a patient with poor biochemical control who is unable (due to busy lifestyle) to take L-T4 fasted and separate from food/drink?*	35 (37.6)	50 (53.8)	5 (5.4)	3 (3.2)
B9- Which of the following preparations of L-T4 would you prescribe for a patient established on L-T4 tablets who has good biochemical control of hypothyroidism but continues to have symptoms?*	66 (71.0)	23 (24.7)	4 (4.3)	0 (0)

*In all of the above scenarios choices for soft-gel capsules + liquid solutions were significantly fewer than other options (p < 0.001).

### Monitoring of the Treatment With Thyroid Hormone

After starting L-T4 treatment, 61 (65.6%) of those who responded to this question recommended checking the TSH after 4-6 weeks and 32 (34.4%) after 8 weeks (4-6 weeks vs. 8 weeks; p=0.003). None suggested control after 2 weeks or relying mostly on clinical evaluation. After switching to a different formulation or changing from one manufacturer’s L-T4 tablets to another’s, 48 (51.6%) would re-check TSH after 4-6 weeks and 34 (36.6%) after 8 weeks (4-6 weeks vs. 8 weeks; p=0.12). Six (6.5%) did not think it was necessary to check TSH if the dosage was the same and five (5.4%) would rely on clinical evaluation only.

### Treatment of Euthyroid Patients With Thyroid Hormones

**Figure 1:**
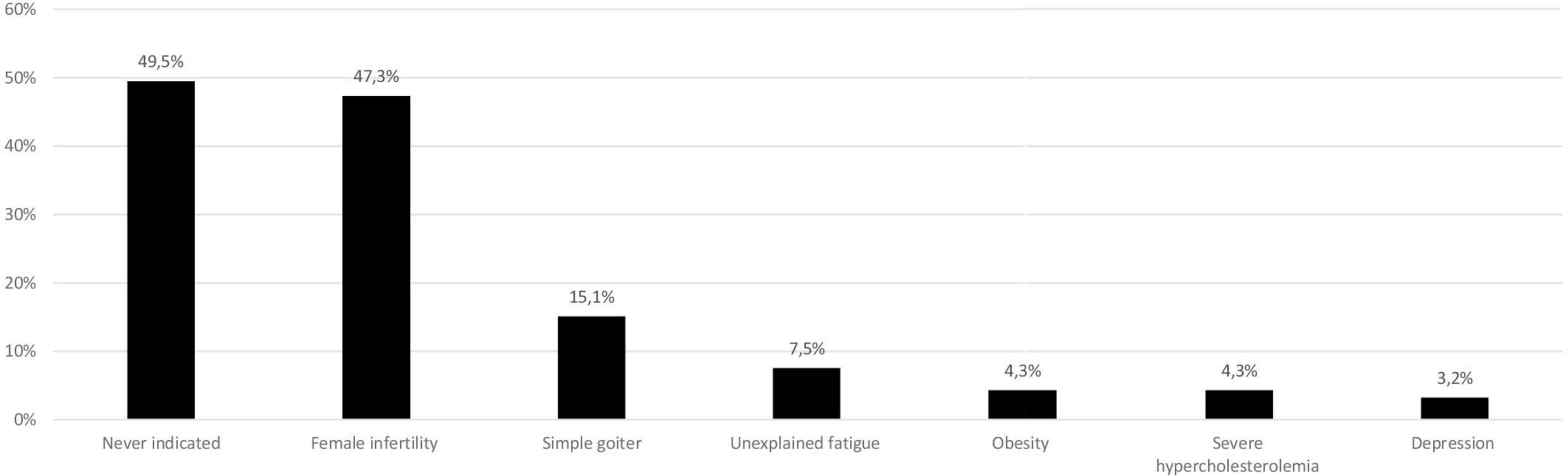
Use of thyroid hormones in euthyroid subjects. Total is >100% as responders were allowed to tick more than one options in the questionnaire (data from 93 respondents, 23 did not answer this question). The same 23 individuals did not respond in **Figures 1**–**3**.

Participants were asked to respond whether thyroid hormones may be indicated in biochemically euthyroid patients with: unexplained fatigue; obesity resistant to life-style interventions; severe hypercholesterolemia, as a complementary treatment; depression resistant to anti-depressant medications; female infertility with high level of thyroid antibodies; simple goiter growing over time; or whether treatment is never indicated for these patients. Of those who answered the question, in biochemically euthyroid patients, 46 (49.5%) indicated that treatment with thyroid hormones was never indicated, 44 (47.3%) would consider treatment in case of female infertility with high levels of thyroid peroxidase (TPO) antibodies, and 14 (15.1%) in case of simple goiter growing over time. Only a minority would consider treatment for unexplained fatigue (7.5%), obesity resistant to lifestyle interventions (4.3%), as a complementary treatment for severe hypercholesterolemia (4.3%), or depression resistant to antidepressants (3.2%) (never use vs. use; p=0.92) (**Figure 1**).

### Use of Combination Therapy With L-T4 and L-T3

**Figure 2:**
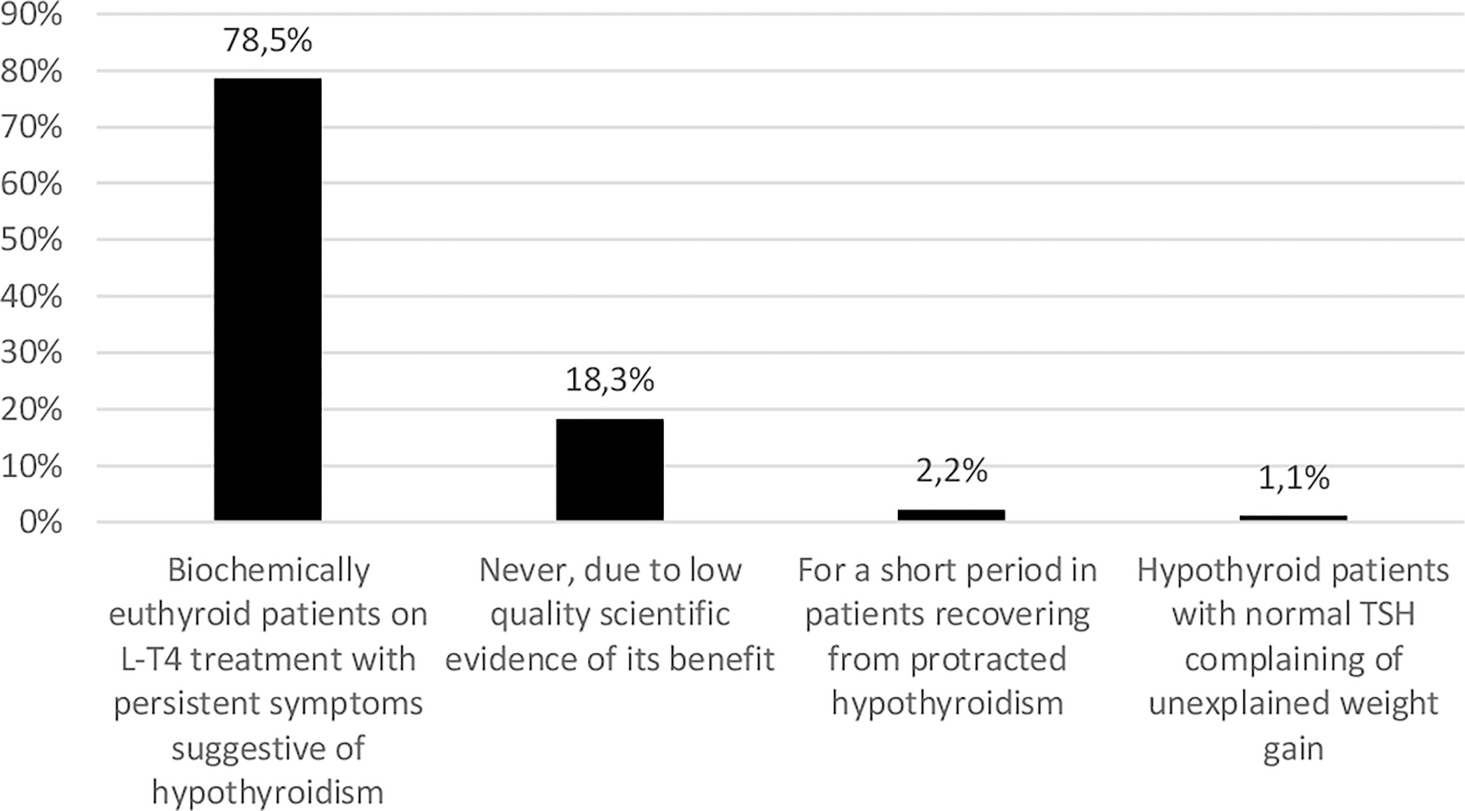
Use of levothyroxine (L-T4) and liothyronine (L-T3) combination therapy in patients with hypothyroidism (data from 93 respondents, 23 did not answer this question). The same 23 individuals did not respond in **Figures 1**–**3**.

The majority of respondents who answered this question (73; 78.5%) considered combination therapy in biochemically euthyroid patients on L-T4 treatment with persistent symptoms suggestive of hypothyroidism. Seventeen (18.3%) would never use combination therapy due to low quality scientific evidence for benefit. Two (2.2%) would consider combination therapy for a short period in patients recovering from protracted hypothyroidism, while one (1.1%) would suggest it in hypothyroid patients with normal TSH complaining of unexplained weight gain (combination therapy vs. never use; p<0.001). (**Figure 2**).

### Persistent Symptoms in L-T4 Treated Patients

**Figure 3:**
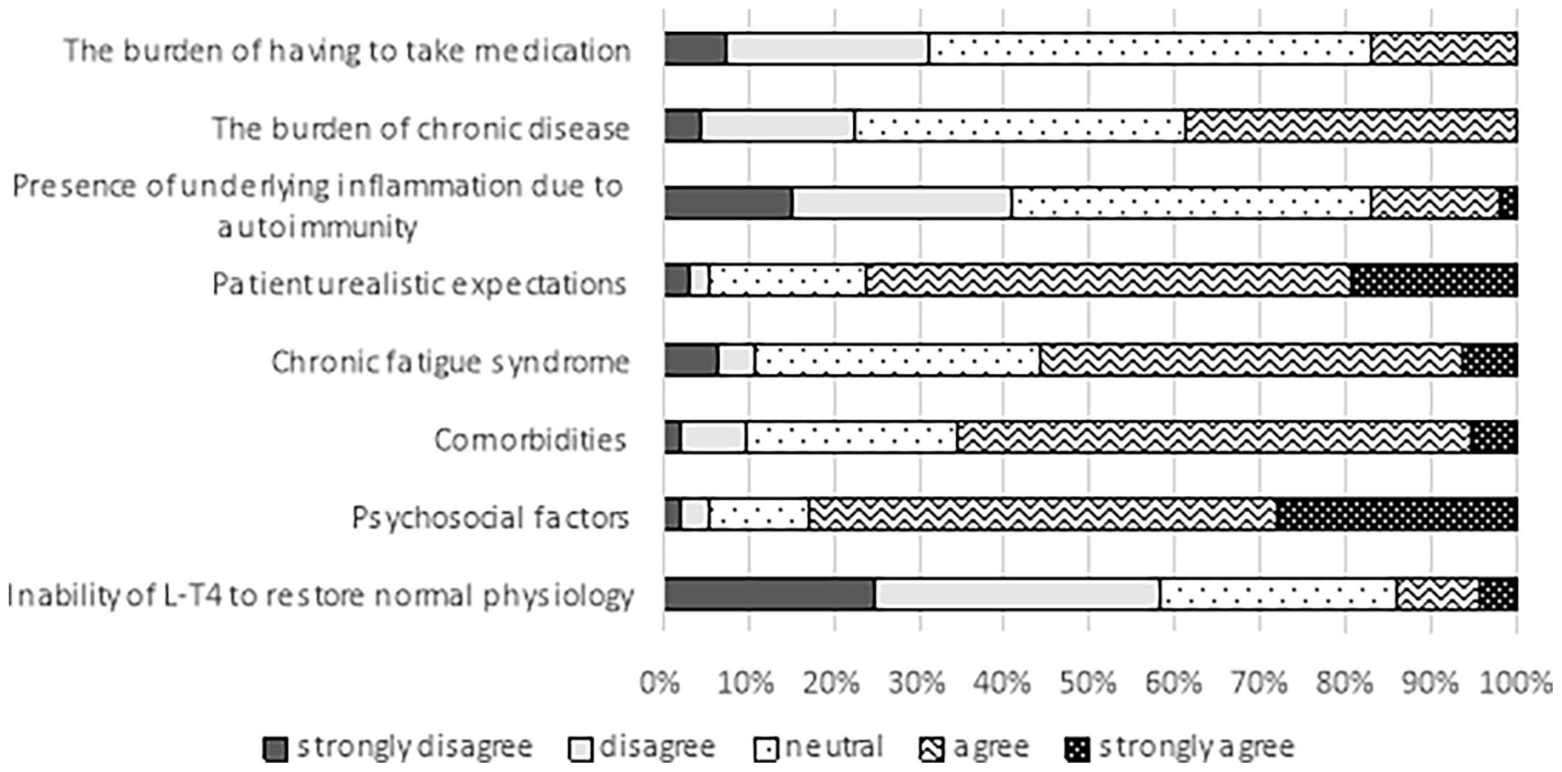
Possible factors explaining persistent hypothyroid symptoms despite biochemical euthyroidism in patients treated with L-T4 (data from 93 respondents, 23 did not answer this question). The same 23 individuals did not respond in **Figures 1**–**3**.

Thirty-four (35.6%) respondents to this question indicated that persistent symptoms despite normal serum TSH were observed in their practice in 6-10% of patients, 22 (23.7%) in 11-30% of patients, 21 (22.6%) in less than 5% of patients, 5 (5.4%) in more than 30% patients, and 11 (11.8%) were not sure (**Table 3**). As for the trend over the past five years, 54 (58.1%) respondents saw more such cases, 24 (25.8%) experienced no change, three (3.2%) responded fewer, and eleven (11.8%) were not sure (**Table 3**). Only a minority (14%) responded that persistent symptoms might be due to L-T4’s inability to restore normal physiology while the majority agreed that the cause could be patients’ unrealistic expectations (76.4%), psychosocial factors (82.8%), or comorbidities (65.6%). Many (56%) also suspected chronic fatigue syndrome (**Figure 3**).

**Table 3:** Respondents’ (n = 93) perceptions about persistence of hypothyroid symptoms despite normal serum TSH. 23 did not answer this question.

Physicians’ perception	Response	N (%)
Frequency of persistent symptoms despite normal serum TSH	>30%	5 (5.4)
	11-30%	22 (23.7)
	6-10%	34 (35.6)
	<5%	21 (22.6)
	Not sure	11 (11.8)
Trend in the past five years	More such cases	54 (58.1)
	No change	24 (25.8)
	Fewer	3 (3.2)
	Not sure	11 (11.8)

### Use of Dietary Supplements

Of those who answered the question, 33 (35.5%) would use dietary supplements such as selenium and iodine upon request from the patient or as a complementary treatment; four (4.3%) would suggest use in case of coexisting autoimmune thyroiditis; none for subclinical hypothyroidism, while 56 (60.2%) answered that they should never be used (use vs. never use; p=0.049).

### Endocrinologists With Hypothyroidism

Seven (7.5%) participants had hypothyroidism. One (1.1%) reported excessive tiredness and the same respondent tried DTE treatment, which led to overtreatment. One respondent had tried combination of L-T4 and L-T3 but did not experience any improvement in well-being and switched back to L-T4. Seventy-two (78.3%) of those who answered the question would not consider combination therapy with L-T4 and L-T3 for themselves if they were to develop hypothyroidism, whereas 20 (21.7%) would consider this option (would consider vs. would not consider combination therapy; p<0.001).

## Discussion

This study, first of its kind in the Swedish medical literature, summarizes the opinions of 116 Swedish medical specialists, mostly endocrinologists, on different aspects of treatment of hypothyroidism.

### Response Rate

The Swedish response rate (28.2%) was slightly lower than the average of the other published national THESIS studies (33%) which has so far varied between 25.8% (Spain) (28) and 54.4% (Poland) (27). The fact that both the invitation email and the reminder were sent at the time of the peaking second wave of the covid-19 pandemic in Sweden offers a possible explanation. Additionally, some of the questions, such as those regarding different L-T4 formulations, might have been considered irrelevant as these formulations are rarely used in Sweden. Moreover, and importantly, most hypothyroid patients in Sweden are treated in the primary care setting.

### Treatment of Hypothyroid and Euthyroid Patients With L-T4

In accordance with international guidelines (30, 31) and with the results of all the national THESIS studies published so far (24–29), L-T4 was the treatment of choice for the Swedish endocrinologists. The majority of Swedish endocrinologist preferred L-T4 tablets and did not expect any major effect of switching to different formulations such as soft-gel capsules or liquid L-T4, or tablets from a different manufacturer, in situations such as concomitant ingestion of interfering drugs, intolerance to various foods, unexplained poor biochemical control, poor biochemical control due to inability to take L-T4 separate from food/drink, or persistent symptoms despite good biochemical control. This is in line with the results of the Danish (29), Polish (27), Bulgarian (25), Romanian (26), and Spanish (28) studies but in sharp contrast to the Italian study (24). The vast majority of the Italian endocrinologist would recommend soft-gel capsules or liquid L-T4 solutions in all these scenarios except for “persistent symptoms despite good biochemical control”. This may be because liquid L-T4 and/or soft-gel capsules were not widely available outside of Italy at time of survey. Moreover, most studies on soft-gel capsules have been performed in Italy and the products have been available in the Italian market for a longer time than in other countries, rendering the Italian physicians more familiar with these products. Additionally, the requirement for case-by-case approval by the national medical products agencies possibly makes physicians more reluctant to prescribe these formulations. Finally, the scientific evidence favoring use of soft-gel capsules and liquid preparation over L-T4 tablets needs to be tested in large-scale randomized studies, as recently reviewed by Nagy et al. (4).

Swedish respondents did not recognize unexplained fatigue, obesity, severe hypercholesterolemia, or depression as indications for thyroid hormone treatment, which is in accordance with current guidelines (30, 31). Interestingly, 49.5% reported that treatment with thyroid hormone was never indicated for biochemically euthyroid patients, while almost the same proportion (47.3%) of the Swedish respondents would consider L-T4 treatment in case of high levels of TPO-antibodies and female infertility. Similar proportions of Romanian (36.4%) (26), Italian (37.3%) (24), Danish (42.1%) (29) and Spanish (48.5%) (28) and even a larger proportion of the Polish (63.4%) (27) respondents indicated that they would do likewise. Anti-TPO positivity has been associated with increased risk of miscarriage (32) and adverse obstetrical outcomes (33, 34), however, recent evidence has not supported these findings (35). According to the latest guidelines of the American Thyroid Association (ATA) (36) and guidelines of the Swedish Society of Obstetrics and Gynecology (SFOG) (37), treatment with L-T4 might be considered in euthyroid anti-TPO positive women with TSH within the upper normal reference range (2.5-4.0 mIU/L), especially in those with a history of miscarriage or undergoing fertility treatment. The rationale is to secure euthyroidism in case of pregnancy, although current evidence from randomized trials (38, 39) suggests that L-T4 treatment neither increases fertility rates nor reduces the risk of adverse pregnancy outcomes in these individuals.

Fourteen percent of the respondents recommended L-T4 treatment in euthyroid individuals with simple goiter growing over time, a rate similar to their Danish (12.5%) (29) and Italian (18%) (24) colleagues, while Spanish (21.2%) (28), Romanian (24%) (26), Polish (40.3%) (27), and Bulgarian (55%) (25) physicians would recommend it even more frequently. This practice is against current guidelines, based on modest effects and significant risks associated with TSH suppression, such as osteoporosis (40), cardiovascular morbidity (41), and increased mortality (42). Ongoing use of L-T4 in simple goiter growing over time probably represents the European endocrinologists´ reluctance to abandon old practices despite long-established evidence (43).

### Use of Combination Therapy With L-T4 and L-T3

Combination treatment with L-T3 + L-T4 is recommended by neither the European nor the American Guidelines (2, 31) on hypothyroidism, based on insufficient evidence of its superiority over L-T4 treatment and lack of long-term data. According to the European guidelines, L-T4 + L-T3 combination therapy might be considered as a trial in L-T4-treated patients who have persistent complaints despite biochemical euthyroidism after exclusion of other autoimmune diseases and comorbidities (2), the latter being very prevalent in patients with hypothyroidism (44, 45). While the vast majority considered L-T4 the initial treatment of choice for hypothyroidism, 78.5% of the respondents would prescribe L-T4 + L-T3 combination therapy in the presence of persistent symptoms suggestive of hypothyroidism. This number was similar to the results of the Danish (71%) (29) but a lot higher than in the Italian (40%) (24), Romanian (35.9%) (26), and Polish (32.2%) (27) surveys. A survey by the American Thyroid Association revealed similar findings to ours, with a high percentage of physicians willing to prescribe combination therapy under specific circumstances such as persisting hypothyroid symptoms (46). These results suggest that this topic is still highly controversial and balancing patient dissatisfaction and demands and lack of scientific evidence remains a challenge. Well-designed randomized clinical trials in patients with persistent symptoms after exclusion of comorbidities are required to address this issue. Although our recent large register-based study (21) provided reassuring evidence regarding the risk of cancer and mortality in L-T3 treated Swedish patients, there could still be other health risks associated with long-term L-T3 therapy warranting randomized trials examining the safety of the combined treatment. Moreover, the cost-effectiveness of the combined treatment also remains an issue, as it is more expensive than L-T4 treatment alone (47) and usually requires more frequent monitoring.

As to the cause of patients´ dissatisfaction with L-T4 treatment, the majority (86%) stated other factors than the inability of L-T4 to achieve tissue euthyroidism. DTE treatment was not popular among Swedish physicians, probably due to limited scientific evidence (22) and the need of approval by the Swedish Medical Products Agency. Interestingly, while 78.5% of the respondents would prescribe L-T4 + L-T3 combination therapy to their patients in the presence of persistent symptoms suggestive of hypothyroidism, the same proportion (78.3%) would not consider combination therapy for themselves should they develop hypothyroidism. This potential discrepancy can be better explored from the aggregate data of all 28 countries that contributed to THESIS. However, it is of interest that in all national surveys so far published, the proportion of physicians who would consider combination therapy for themselves was lower than for their patients.

### Use of Dietary Supplements

More than a half (60.2%) of the respondents suggested that dietary supplements, including selenium and iodine, should never be used. Approximately one third suggested that they could be used upon request from the patient or as a complementary treatment, while only few recommended them in the context of coexisting autoimmune thyroiditis. This result is probably based on insufficient evidence for clinical efficacy of selenium supplementation (48), despite evidence of an effect on TPOAb concentrations (49), and the fact that the Swedish population is considered iodine sufficient from iodine fortification of salt since 1936 (50, 51).

### Strengths and Limitations

All of the participants being medical specialists and nearly all of them endocrinologists is a strength, as they represent key opinion leaders. While one can assume that endocrinologists’ practrices may reflect choices made by the physicians in general practice, where the majority of patients are treated, direct evidence supporting this is lacking. The main limitation is the low response rate, which questions whether the conclusions of the study can be considered representative for all Swedish endocrinologists. However, it is likely that most of the non-responders were not clinically active, or did not treat patients with hypothyroidism thus leading to a falsely low response rate.

In conclusion, L-T4 tablets were the primary treatment of choice for hypothyroidism among Swedish endocrinologists. In general, alternative L-T4 formulations, like soft-gel capsules and liquid solutions, were not recommended. The majority would consider L-T3 + L-T4 combination treatment for patients with a diagnosis of hypothyroidism who are biochemically euthyroid on L-T4 and complain of persistent hypothyroid symptoms. In a biochemically euthyroid patient, the only scenario where L-T4 was considered by a significant number of participants was female infertility with high TPO antibody levels. This deviation from endocrine society guidelines merits further study. Overall, the responses of the Swedish endocrinologists did not deviate from the recommendations of the current international guidelines and were very similar to the responses of their Danish colleagues (29). Compared with the other national THESIS studies, country-specific factors such as the availability and cost of various preparations and local traditions most probably influenced the choice of treatment. In the future, the joint analysis of the entire THESIS cohort summarizing data from 28 European countries will provide us with a more universal view of this topic.

## Data Availability Statement

The raw data supporting the conclusions of this article will be made available by the authors, without undue reservation.

## Author Contributions

PP, EP, RA, EN, and LH contributed to the study conception, design and creation of the survey. TP and ML were responsible for writing of the invitation letter and reminder. TP was responsible for data analysis. TP wrote the first draft of the manuscript and all authors commented on previous versions of the manuscript. All authors contributed to the article and approved the submitted version.

## Funding

The first author was supported by grants from Lundgrens stiftelse and SUS fonder. The THESIS study received funding from IBSA, Institute Biochimique SA. The funder was not involved in the study design, collection, analysis, interpretation of data, the writing of this article, or the decision to submit it for publication. All authors declare no other competing interests.

## Conflict of Interest

EN, EP, PP, and LH have undertaken consultancy work for IBSA.

The remaining authors declare that the research was conducted in the absence of any commercial or financial relationships that could be construed as a potential conflict of interest.

IBSA had no role in the design of the survey, data analysis, data presentation, data interpretation or writing the manuscript; the authors did not receive remuneration by IBSA.

## Publisher’s Note

All claims expressed in this article are solely those of the authors and do not necessarily represent those of their affiliated organizations, or those of the publisher, the editors and the reviewers. Any product that may be evaluated in this article, or claim that may be made by its manufacturer, is not guaranteed or endorsed by the publisher.

## Abbreviations

THESIS, Treatment of Hypothyroidism in Europe by Specialists: An International Survey.
